# RPEMHC: improved prediction of MHC–peptide binding affinity by a deep learning approach based on residue–residue pair encoding

**DOI:** 10.1093/bioinformatics/btad785

**Published:** 2024-01-04

**Authors:** Xuejiao Wang, Tingfang Wu, Yelu Jiang, Taoning Chen, Deng Pan, Zhi Jin, Jingxin Xie, Lijun Quan, Qiang Lyu

**Affiliations:** School of Computer Science and Technology, Soochow University, Suzhou, Jiangsu 215006, China; School of Computer Science and Technology, Soochow University, Suzhou, Jiangsu 215006, China; Province Key Lab for Information Processing Technologies, Soochow University, Suzhou, Jiangsu 215006, China; Collaborative Innovation Center of Novel Software Technology and Industrialization, Nanjing, Jiangsu 210000, China; School of Computer Science and Technology, Soochow University, Suzhou, Jiangsu 215006, China; School of Computer Science and Technology, Soochow University, Suzhou, Jiangsu 215006, China; School of Computer Science and Technology, Soochow University, Suzhou, Jiangsu 215006, China; School of Computer Science and Technology, Soochow University, Suzhou, Jiangsu 215006, China; School of Computer Science and Technology, Soochow University, Suzhou, Jiangsu 215006, China; School of Computer Science and Technology, Soochow University, Suzhou, Jiangsu 215006, China; Province Key Lab for Information Processing Technologies, Soochow University, Suzhou, Jiangsu 215006, China; Collaborative Innovation Center of Novel Software Technology and Industrialization, Nanjing, Jiangsu 210000, China; School of Computer Science and Technology, Soochow University, Suzhou, Jiangsu 215006, China; Province Key Lab for Information Processing Technologies, Soochow University, Suzhou, Jiangsu 215006, China; Collaborative Innovation Center of Novel Software Technology and Industrialization, Nanjing, Jiangsu 210000, China

## Abstract

**Motivation:**

Binding of peptides to major histocompatibility complex (MHC) molecules plays a crucial role in triggering T cell recognition mechanisms essential for immune response. Accurate prediction of MHC**–**peptide binding is vital for the development of cancer therapeutic vaccines. While recent deep learning-based methods have achieved significant performance in predicting MHC**–**peptide binding affinity, most of them separately encode MHC molecules and peptides as inputs, potentially overlooking critical interaction information between the two.

**Results:**

In this work, we propose RPEMHC, a new deep learning approach based on residue–residue pair encoding to predict the binding affinity between peptides and MHC, which encode an MHC molecule and a peptide as a residue–residue pair map. We evaluate the performance of RPEMHC on various MHC-II-related datasets for MHC**–**peptide binding prediction, demonstrating that RPEMHC achieves better or comparable performance against other state-of-the-art baselines. Moreover, we further construct experiments on MHC-I-related datasets, and experimental results demonstrate that our method can work on both two MHC classes. These extensive validations have manifested that RPEMHC is an effective tool for studying MHC–peptide interactions and can potentially facilitate the vaccine development.

**Availability:**

The source code of the method along with trained models is freely available at https://github.com/lennylv/RPEMHC.

## 1 Introduction

T-cell-mediated adaptive immunity is a specific type of immunity that the body acquires to adapt to its living environment and recognize foreign antigens (Cook 2000). Central to this process is the major histocompatibility complex (MHC) molecule, which recognizes antigens and determines whether an immune response occurs. MHC molecules bind to antigenic peptides and present them on the surface of antigen-presenting cells (APCs) to stimulate an immune response that eliminates foreign pathogens (Al-Daccak *et al.* 2004). Consequently, accurate identification of peptides binding to MHC molecules is crucial for vaccine design and immunotherapy (Hu *et al.* 2018).

There are two major classes of MHC molecules: class I (MHC-I) and class II (MHC-II) (Maenaka and Jones 1999), and both classes exhibit peptide presentation specificities. The peptide binding groove of MHC-I molecules is mainly associated with closed ends, while the binding groove of MHC-II molecules has more open ends. Therefore, MHC-I molecules bind peptides with relatively stable lengths (mostly 8–11), whereas MHC-II molecules bind peptides with a wider range of lengths (mostly 13–25) (Chicz *et al.* 1992), resulting in precise identification of peptides binding to MHC-II much more challenging than that of MHC-I.

Traditional experimental methods for identifying effective MHC-binding peptides are time-consuming and costly, given that only a small fraction of viral protein-derived short peptides can bind to MHC molecules. With recent advancements in MHC**–**peptide binding databases and computational resources, a multitude of efficient computational methods have been developed to narrow down the range of MHC-binding peptides. These methods can be divided into two categories: allele-specific and pan-specific ones. Specifically, the allele-specific methods train a separate model for each MHC allele and they just make prediction of individual MHC alleles (Zhang *et al.* 2012), such as NetMHC (Lundegaard *et al.* 2008, Andreatta and Nielsen 2016). On the other hand, the prominent feature of pan-specific methods are capable of making binding affinity prediction for not only the alleles that are present in the training data, but also new alleles that are unseen in the training data (Garde *et al.* 2019, Liu *et al.* 2021, Chu *et al.* 2022, Wang *et al.* 2022). The pan-specific methods pool the binding data of different MHC alleles together as input and train a general model for all alleles. For example, the NetMHCpan (Nielsen and Andreatta 2016, Jurtz *et al.* 2017) and NetMHCIIpan (Andreatta *et al.* 2015, Jensen *et al.* 2018, Reynisson *et al.* 2020b) toolkit based on shallow neural networks are widely used pan-specific methods to predict peptides binding to MHC-I and MHC-II molecules, respectively. However, the performance of such methods is limited due to their simple models difficult to learn complex patterns from the data. To this end, deep learning-based methods have been developed for MHC**–**peptide binding prediction, such as PUFFIN (Zeng and Gifford 2019), MHCAttnNet (Venkatesh *et al.* 2020), BERTMHC (Cheng *et al.* 2021), DeepMHCII (You *et al.* 2022). For instance, the DeepMHCII method employs deep convolutional neural networks (CNNs) and is the state-of-the-art one to predict MHC**–**peptide binding affinity for MHC-II (You *et al.* 2022); the MHCAttnNet method uses bidirectional long short-term memory (Bi-LSTM) and the attention mechanism and is the state-of-the-art one to predict MHC**–**peptide binding for both MHC-I and MHC-II (Venkatesh *et al.* 2020). Despite advanced deep learning techniques used, most of the existing methods usually encode the sequences of MHC molecule and peptide separately as the input, which makes them potentially overlook critical interaction information between them, resulting in performance improvement restriction.

In this work, we develop a new deep learning-based approach based on residue–residue pair encoding, referred to as RPEMHC, for the pan-specific prediction of MHC-II and peptide binding affinity. More specifically, in order to take advantage of the critical interaction information between MHC molecules and peptides, RPEMHC encodes a pair of an MHC-II and a peptide as a residue–residue interaction matrix, in which the height represents the 34 amino acids in the MHC pseudo-sequence, the width represents the 20 amino acids in the peptide, and each element represents an amino acid match in different positions of the corresponding sequences of MHC and peptide. The architecture of RPEMHC consists of superimposed CNN and LSTM layers, by which the local and global features of MHC–peptide binding are learned and then integrated to capture its intrinsic patterns. The performance of RPEMHC in the prediction of binding affinity between MHC-II molecules and peptides has been evaluated on a variety of benchmark datasets under different experimental settings, including five-fold cross-validation, leave-out-one molecule (LOMO), and independent tests. Experimental results on five-fold cross-validation, leave-out-one molecule (LOMO), and two independent tests of binding affinity data have demonstrated that RPEMHC achieves improved performance against other state-of-the-art baseline methods, such as NetMHCIIpan-3.2 (Jensen *et al.* 2018), PUFFIN (Zeng and Gifford 2019), DeepMHCII (You *et al.* 2022), and NetMHCIIpan-4.0 (Reynisson *et al.* 2020b), while on T-cell epitope benchmark RPEMHC achieves better or comparable performance against NetMHCIIpan-3.2 (Jensen *et al.* 2018), DeepMHCII (You *et al.* 2022), and NetMHCIIpan-4.0 (Reynisson *et al.* 2020b). In addition, in order to demonstrate the general applicability of the RPEMHC model, in the sense that the model architecture can work on both MHC-I and MHC-II molecules, the performance of RPEMHC has been further validated on three benchmark datasets related to MHC-I molecules. The results indicate that RPEMHC can be generalized well on the prediction of MHC-I–peptide binding. Based on these extensive validations, it can be concluded that RPEMHC is an effective tool for examining MHC–peptide interactions and can potentially facilitate the vaccine development.

## 2 Materials and methods

### 2.1 Datasets

Various widely used benchmark datasets are used to evaluate the performance of RPEMHC and comparative methods, including IEDB2016 (Jensen *et al.* 2018), IC50${}_{\mathrm{test}}$ (Cheng *et al.* 2021), Binary${}_{\mathrm{test}}$ (You *et al.* 2022), T-cell epitope benchmark (Jensen *et al.* 2018, Reynisson *et al.* 2020b), BC2015 (Jensen *et al.* 2018), MHC-I_2020_ (Venkatesh *et al.* 2020), MHC-II_2020_ (Venkatesh *et al.* 2020), MHC-I_2015_ (Nielsen and Andreatta 2016), and CD8 epitope benchmark (Jurtz *et al.* 2017).

**IEDB2016**. IEDB2016 was collected from the Immune Epitope Database (IEDB) (Jensen *et al.* 2018) up to 2016 and contains 134281 data entries of MHC–peptide binding affinity covering 80 types of MHC-II molecules, which include 36 HLA-DR, 27 HLA-DQ, 9 HLA-DP, and 8 H-2 molecules. The affinity values in IEDB2016 were transformed from IC50 to values between 0 and 1 by the formula 1−log(IC50)/log(50000).

**IC50** ${}_{\mathrm{test}}$ **& Binary** ${}_{\mathrm{test}}$ . These two independent datasets are used for testing the generalizability of RPEMHC. Specifically, the IC50${}_{\mathrm{test}}$ dataset contains 2413 data entries of MHC-II and peptide binding affinity (Cheng *et al.* 2021) covering 47 types of MHC-II molecules. The Binary${}_{\mathrm{test}}$ dataset is a binary classification one, which consists of 639 binding samples and 218 non-binding samples for 10 types of HLA-DB molecules with 857 peptides (You *et al.* 2022).

**T-cell epitope benchmark**. This independent dataset is also used for evaluating the generalization capability of RPEMHC and consists of 2167 MHC-II restricted T-cell epitopes, which combine the epitope data from NetMHCIIpan-3.2 (Jensen *et al.* 2018) and NetMHCIIpan-4.0 (Reynisson *et al.* 2020b) by excluding the overlapping epitopes.

**BC2015**. This dataset is used to identify the binding core of an MHC-II peptide complexes, which consists of 51 complexes from PDB (Jensen *et al.* 2018).

**MHC-I_2020_ & MHC-II_2020_.** MHC-I_2020_ and MHC-II_2020_ are used to further evaluate the general applicability of RPEMHC architecture and are two binary classification datasets of MHC-I–peptide binding and MHC-II–peptide binding, respectively. The MHC-I_2020_ dataset contains 491018 MHC–peptide data entries covering 161 types of MHC-I molecules, which contains 379783 binding samples and 111235 non-binding samples. The MHC-II_2020_ dataset contains 64954 MHC–peptide data entries over 49 types of MHC-II molecules, which contains 36035 binding samples and 28919 non-binding samples.

**MHC-I_2015_.** MHC-I_2015_ is a dataset of MHC-I and peptide binding affinity, which was used to evaluate the performance of NetMHCpan-3.0 specifically designed for MHC-I molecules (Nielsen and Andreatta 2016). This dataset consists of 186,684 MHC-I–peptide binding affinity measurements as positive samples covering 172 types of MHC-I molecules, and 14601 negative samples that were randomly generated (Nielsen and Andreatta 2016). Notably, these random negative samples are only used to train the model and are excluded from all evaluations.

**CD4 epitope benchmark & CD8 epitope benchmark**. CD4 epitope benchmark is an independent test set of MHC-II restricted CD4+ epitopes obtained from Reynisson et al. (Reynisson *et al.* 2020a), and is refined to contain 917 epitopes restricted to 20 types of MHC-II molecules in NeMHCIIpan-4.0 (Reynisson *et al.* 2020b). CD8 epitope benchmark is an independent test set of MHC-I restricted CD8+ epitopes obtained from Jurtz et al. (Jurtz *et al.* 2017) and the IEDB and is refined to contain 1660 epitopes restricted to 52 types of MHC-I molecules in NetMHCpan-4.1 (Reynisson *et al.* 2020b).

### 2.2 Problem formulation

Given the amino acid sequences of a pair of MHC molecule and peptide, the task is a regression problem to predict the binding affinity between them. Here, the sequence of an MHC molecule of length *L* is represented as $S_{\mathrm{MHC}}=\{s_{1},s_{2},\ldots ,s_{L}\}$, where each $s_{i}$ represents one of the 20 amino acids, 1≤i≤L. Similarly, the sequence of a peptide of length $L^{\prime }$ is represented as $S_{\mathrm{pep}}=\{s_{1},s_{2},\ldots ,s_{L^{\prime }}\}$. Note that the sequence of an MHC molecule is usually simplified to a pseudo-sequence of length 34, i.e. it is a non-contiguous subsequence of the original sequence (Karosiene *et al.* 2013, You *et al.* 2022). This MHC pseudo-sequence extracts amino acid residues, which are considered to be essential for the MHC–peptide binding (Karosiene *et al.* 2013), and consists of 15 residues in the alpha chain and 19 residues in the beta chain of the MHC molecule. In addition, since the length of most peptide sequences is less than 20, the length of all peptide sequences is padded or truncated to the length of 20, so as to maintain the consistency of the input dimension. Therefore, *L* and $L^{\prime }$ have values of 34 and 20, respectively.

### 2.3 The model architecture of RPEMHC

**Figure 1.**
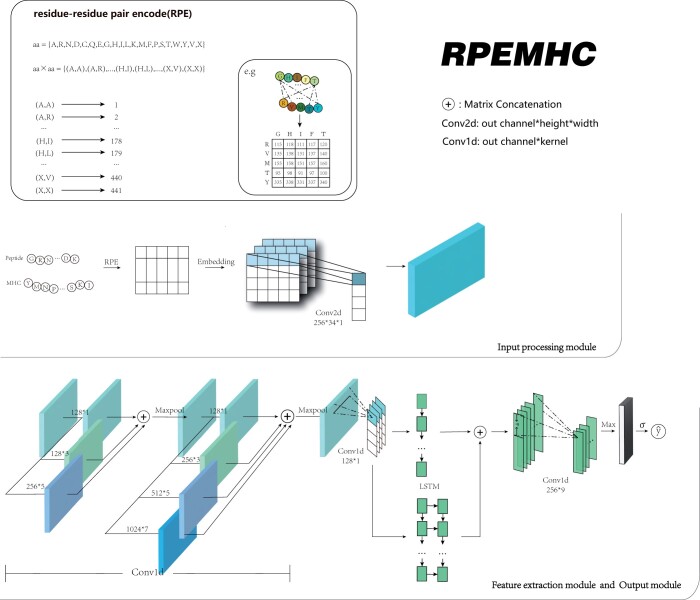
The model architecture of RPEMHC. RPE in the input processing module is used to encode each MHC–peptide sequence into an interaction matrix. The feature extraction module and the output module are used to extract latent representation with multi-level CNNs and produce the final output, respectively. Convolution modules with different colors represent convolution kernels with different scales. The notation m*n besides the arrow indicates the number of kernels *m* and the size of kernel *n*.

The model architecture of the proposed RPEMHC is depicted in Fig. 1, which is composed of an input processing module, a feature extraction module, and an output module. In the following, we will describe these three modules in detail.

#### 2.3.1 Input processing module

When faced with the task of MHC–peptide binding prediction, most of existing deep learning approaches usually treated the MHC molecule sequence and the peptide sequence as two separate inputs and processed them separately. However, such separate processing approach may lose the critical interaction information between MHC molecules and peptides, which is valuable for binding prediction. To overcome this problem, we leveraged pairwise residue map to encode the overall interaction between an MHC molecule and a peptide, as shown in Fig. 1.

Specifically, let *A* be a set consisting of 20 types of amino acids and a type of the unknown residues *X*, thus—A—=21, we can create the vocabulary of residue–residue pairs $V_{\mathrm{rcp}}$ as the following equation:

(1) $$V_{\mathrm{rcp}}=\{\langle a,b\rangle |a,b\in A\}.$$

Let *T* be a set of integers which is defined as:

(2) $$T=\{k\in Z^{+}|k\leq |V_{\mathrm{rcp}}|\}.$$

We can define a bijective mapping function from $V_{\mathrm{rcp}}$ to *T*, i.e. $f:V_{\mathrm{rcp}}\to T$. In this way, we can encode an MHC molecule sequence $S_{\mathrm{MHC}}$ and a peptide sequence $S_{\mathrm{pep}}$ into a residue–residue interaction matrix *M* of size $L\times L^{\prime }$, i.e. 34×20, each of which represents the interaction between one residue in the MHC molecule sequence and one residue in the peptide sequence. More precisely, the value of each element $M_{i,j}$, 1≤i≤34, 1≤j≤20, is determined by using the mapping function *f* as follows:

(3) $$M_{ij}=\{f(\langle s_{i},s_{j}\rangle )|s_{i}\in S_{\mathrm{MHC}},s_{j}\in S_{\mathrm{pep}}\}.$$

Note that each residue pair $\langle s_{i},s_{j}\rangle$ represents one residue in the MHC molecule sequence and one residue in the peptide sequence, and thus 〈a,b〉 and 〈b,a〉 in $V_{\mathrm{rcp}}$ are mapped to different integers in *T*.

Upon the residue–residue interaction matrix *M* obtained, it was fed into the embedding layer to obtain a feature representation $M^{\prime }$, whose size is (34,20,Embedding_dim), following a convolution layer with a kernel size of (34, 1) to fuse all the information of the MHC molecule to each residue in the peptide as follows.

(4) $$I=\mathrm{conv}(M^{\prime }),$$

where the size of *I* is (1,20,Embedding_dim).

Since the residues in an MHC pseudo-sequence are crucial for peptide binding, the above RPE encoding way can fuse the important information of non-contiguous MHC-II sequence into each residue in the contiguous peptide. Due to the high polymorphism of MHC molecules, their sequences are strongly similar. Even if the representation of MHC molecules in the form of pseudo-sequences greatly avoids using long sequences, the high similarities among these sequences may lead to difficulties in recognizing different MHC molecules. In contrast, binding affinity prediction from the aspect of peptides is a potentially good solution. Therefore, RPEMHC fused the MHC molecule information as a whole into each residue of the peptide and predicted the binding affinity with the fused peptide.

#### 2.3.2 Feature extraction module

The feature extraction module consists of three convolution layers Krizhevsky *et al.* (2017); Kim (2014), followed by one layer of LSTM (Hochreiter and Schmidhuber 1997), and followed by one convolution layer finally. Based on the output *I* of the previous input processing module, the feature extraction module can be described by the following equation:

(5) $$F(I)=\mathrm{conv}(\mathrm{lstm}(\mathrm{con}v_{3}(\mathrm{pool}(\mathrm{con}v_{2}(\mathrm{pool}(\mathrm{con}v_{1}(I))))))).$$

Specifically, in the first convolution layer, three convolution blocks with different convolution kernel sizes are used to extract the features from the input *I* in parallel. The kernel sizes of these three convolution blocks are set to 1, 3, and 5, respectively, and the number of convolution kernels is set to 32, 64, and 128, respectively. Different convolution kernels enable a larger field of view so that the information around the residues can be captured in multiple scales. After three convolution blocks of the first convolution layer, three different kinds of feature representation are concatenated and then fed into a max pooling layer for dimensionality reduction, whose kernel size is set to 2. The second convolution layer consists of four parallel convolution blocks with kernel sizes of 1, 3, 5, and 7 and kernel numbers of 128, 256, 512, and 1024, respectively, so as to further extract deeper and multi-scale feature representation. This convolution layer is also followed by a max pooling layer with a kernel size of 3. The third convolution layer consists of one convolution block with a kernel size of 1 and is used to extract higher abstraction features from the concatenated feature maps extracted by the second convolution layer. Therefore, by using these three convolution layers, RPEMHC can extract higher-dimensional abstract features feature representations at different scales.

Although CNNs can make good attention to each part of the input and its surrounding information, they overlook some overall sequence dependence information. LSTM has been demonstrated to be effective in capturing the interdependencies across long sequences (Hochreiter and Schmidhuber 1997). In addition, the combination of CNN followed by LSTM has proven to be an excellent strategy for utilizing the merits of both networks Chollet (2021). Consequently, the CNN layer is connected to two parallel LSTM blocks in the LSTM layer, each of which processes the feature map output of the CNN. These two LSTM blocks consist of one and two LSTM networks, respectively, so that RPEMHC can learn global information in the sequence at different scales. Subsequently, the feature representation extracted by the two LSTM blocks is concatenated and fed into another convolution layer with a kernel size of 9. Since the length of the binding core of peptides is 9, and with that in mind, the kernel size of the final convolution layer is set to 9 to extract the information related to the binding core.

#### 2.3.3 Output module

The output of the feature extraction module is fed into an output module, which is composed of a fully connected layer and an output layer. Assume that a representation *z* of the interaction between an MHC molecule and a peptide is obtained after the fully connected layer. Since the binding affinity labels in our datasets have been converted to values between 0 and 1, i.e. y∈[0,1], by the formula 1−log(IC50)/log(50000), we leverage the output layer to output the predicted binding affinity $\hat{y}\in [0,1]$ as follows.

(6) $$\hat{y}=\sigma (W\cdot z+b),$$

where *W* is the weight and *b* is the bias.

### 2.4 Model training

In this work, RPEMHC was developed to handle the regression problem of MHC–peptide binding affinity prediction, so we here used the mean squared error (MSE) loss function to train our model, which is formally defined as below.

(7) $$\mathrm{MSE}=\frac{1}{N}{\left(y_{i}-\hat{y_{i}}\right)}^{2},$$

where *N* is the total number of samples from the training data, $y_{i}$ is experimentally measured binding affinity of the sample *i*, and $\hat{y_{i}}$ is the predicted binding affinity of the sample *i*. Additionally, to avoid the randomness problem of single model, we trained RPEMHC for twenty times and took the average results of the 20 models as the final prediction value.

### 2.5 Evaluation metrics

In this work, we leveraged the widely used metrics to evaluate the performance of our method and compared with other baseline methods, including the area under the receiver operating characteristics curve (AUC), the area under the precision-recall curve (PRC), Pearson correlation coefficient (PCC), Precision or positive predictive value (PPV), F1-score, and Sensitivity. The definitions of these metrics were provided in Supplementary Text S1. In addition, we formulated the MHC–peptide binding problem as a binary classification task and leveraged the metric AUC to evaluate the prediction performance of PREMHC, so we set a threshold of 500 nM to transform the binding affinity value IC50 to a value of 0 or 1 according to Equation (8).

(8) $$y_{i}=\{\begin{array}{ccc} 0, & y_{i}<1-\log(500)/\log(50000), & \\ 1, & y_{i}>1-\log(500)/\log(50000) & \end{array}$$

## 3 Results and discussion

In this section, we first compared our method RPEMHC against state-of-the-art baseline methods for the prediction of binding affinity between MHC-II and peptides on various benchmarking datasets under different experimental settings. Then, we applied RPEMHC for the binding prediction on both MHC-I and MHC-II datasets to demonstrate its general applicability. Finally, we further provided analysis for the predicted results of RPEMHC.

### 3.1 Performance of RPEMHC on MHC-II–peptide binding affinity prediction

#### 3.1.1 Comparison of RPEMHC with existing methods under five-fold cross-validation on IEDB2016

**Figure 2.**
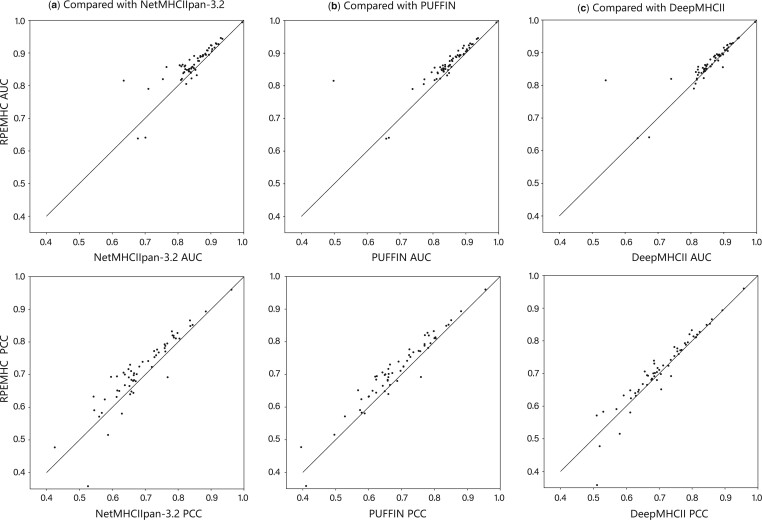
Performance comparison between RPEMHC and (a) NetMHCIIpan-3.2, (b) PUFFIN, and (c) DeepMHCII under five-fold cross-validation on IEDB2016. Each dot represents an MHC-II molecule. The AUC (top) and PCC (bottom) for RPEMHC (y-axis) are compared with the NetMHCIIpan-3.2, PUFFIN, and DeepMHCII (x-axis).

To evaluate the prediction performance of RPEMHC, we performed five-fold cross-validation on the IEDB2016 dataset (the error bounds of cross-validation were provided in Supplementary Table S1), and compared the performance of our method to those of NetMHCIIpan-3.2 (Jensen *et al.* 2018), PUFFIN (Zeng and Gifford 2019), and DeepMHCII (You *et al.* 2022) under the same experimental setting, which had been used for the prediction of MHC-II–peptide binding affinity. The comparison results are shown in Table 1 from two perspectives, i.e. Average and All, where the results of the three compared methods were taken from their corresponding publications. The **Average** indicates the average result of 61 MHC-II molecules each containing more than 40 peptides and at least three binders out of all 80 molecules, and the **All** indicates the result of the whole IEDB2016. As from Table 1, RPEMHC achieved the best prediction results in terms of AUC and PCC on Average and All experiments, as compared to NetMHCIIpan-3.2, PUFFIN, and DeepMHCII. More specifically, as shown in Fig. 2, RPEMHC outperformed NetMHCIIpan-3.2, PUFFIN, and DeepMHCII in terms of AUC on 54, 57, and 49 out of all 61 MHC-II molecules, respectively (paired-samples t-test, *P-*value = $8.293\times 10^{-6}$, $1.194\times 10^{-4}$, $1.716\times 10^{-2}$, respectively), and in terms of PCC on 52, 54, and 47 out of all 61 MHC-II molecules, respectively (paired-samples t-test, *P-*value = $1.405\times 10^{-4}$, $1.438\times 10^{-7}$, $1.821\times 10^{-2}$, respectively). The detailed results of AUC, PCC, and PRC for each MHC-II molecule on IEDB2016 are provided in Supplementary Table S2.

**Table 1.** Comparison of the performance of RPEMHC with NetMHCIIpan-3.2, PUFFIN, and DeepMHCII on IEDB2016 under five-fold cross-validation.^a^

Method	Average		All	
	AUC	PCC	AUC	PCC
NetMHCIIpan-3.2	0.847 (± 0.061)	0.679(± 0.118)	0.873	0.744
PUFFIN	0.846(± 0.072)	0.676(± 0.126)	0.877	0.754
DeepMHCII	0.856(± 0.070)	0.691(± 0.129)	0.882	0.761
RPEMHC	**0.866(** ± **0.057)**	**0.704(** ± **0.118)**	**0.887**	**0.771**

a The best results are indicated in bold. For metrics in the Average setting, the value in parentheses indicates the standard deviation of all MHC-II molecules.

**Figure 3.**
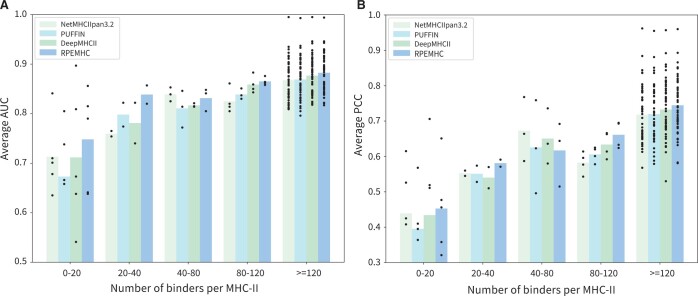
Performance comparison between NetMHCIIpan-3.2, PUFFIN, and DeepMHCII on five categories of MHC-II molecules with different number of binding peptides. Each bar shows the average performance of each method in terms of AUC on all MHC molecules in a certain category. Each scatter point represents the performance of an MHC molecule.

Moreover, in order to evaluate the prediction performance of RPEMHC on MHC-II molecules with a small number of binding peptides, we further analyzed the results of five-fold cross-validation on IEDB2016. We classified the MHC-II molecules into five categories according to their number of binding peptides, i.e. 0–20, 20–40, 40–80, 80–120, and ≥ 120. As shown in Fig. 3, RPEMHC significantly outperformed all three baseline methods for those MHC-II molecules with 0–20 and 20–40 binding peptides. Accordingly, these results demonstrated that RPEMHC can also have superior prediction performance when the amount of data is limited.

#### 3.1.2 Comparison of RPEMHC with existing methods under LOMO on IEDB2016

**Figure 4.**
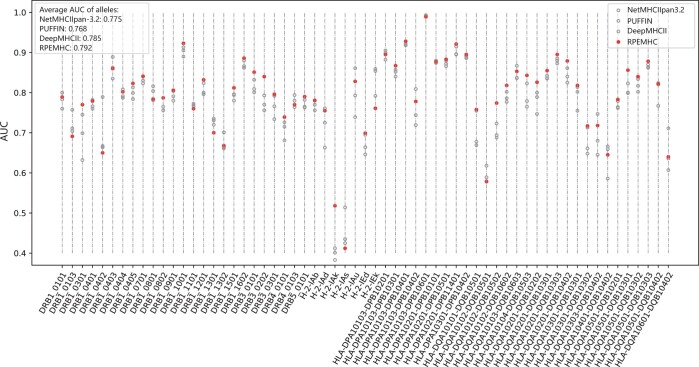
Performance comparison between RPEMHC and NetMHCIIpan-3.2, PUFFIN, and DeepMHCII on IEDB2016 under LOMO experiment. The AUCs for 61 MHC-II molecules for RPEMHC (solid circle) and the other three baseline methods (empty circle).

As the data are continuously updated, the performance for predicting the binding between unknown molecules and peptides is becoming of special significance. To validate the performance of RPEMHC on molecules that have not appeared before, we implemented the leave-one-molecule-out (LOMO) experiments on the IEDB2016 dataset by using the same five-fold cross-validation set-up as above. Specifically, for each MHC-II molecule, the model was trained on four training folds with data points of all other MHC-II molecules from this molecule removed, and tested on the test fold with data points of only this molecule kept, and the out-of-fold predictions from five folds were combined to compute a final LOMO result for each MHC-II molecule. The performance comparison of RPEMHC with NetMHCIIpan-3.2, PUFFIN, and DeepMHCII on 61 MHC-II molecules of LOMO experiment in terms of AUC is shown in Fig. 4 (the detailed results of AUC, PCC, and PRC for each MHC-II molecule are provided in Supplementary Table S3). RPEMHC achieved the highest average AUC of 0.792, which was 0.7% higher than that achieved by the second best method, DeepMHCII. Moreover, RPEMHC outperformed NetMHCIIpan-3.2, PUFFIN, and DeepMHCII in terms of AUC on 46, 50, and 41 out of the 61 MHC-II molecules under LOMO experiment, respectively, all being statistically significant (paired-samples t-test, *P-*value = $1.945\times 10^{-3}$, $3.843\times 10^{-6}$, $1.779\times 10^{-2}$, respectively). Consequently, these results demonstrated the robustness of RPEMHC, namely our method can deal with unknown MHC-II molecules better than other baseline methods.

#### 3.1.3 Independent tests of RPEMHC on IC50${}_{\mathrm{test}}$ and binary${}_{\mathrm{test}}$

In order to demonstrate the generalization capability of RPEMHC, we performed tests on two independent datasets IC50${}_{\mathrm{test}}$ and Binary${}_{\mathrm{test}}$, which were filtered out of the data already used in IEDB2016. We compared the prediction performance of RPEMHC to those of NetMHCIIpan-3.2, PUFFIN, DeepMHCII, and NetMHCIIpan-4.0_BA on these two datasets, where the subscript *BA* of NetMHCIIpan-4.0_BA indicates to select the output of binding affinity (BA) predictions from its web server.

The performance comparison of RPEMHC with NetMHCIIpan-3.2, PUFFIN, DeepMHCII, and NetMHCIIpan-4.0_BA on IC50${}_{\mathrm{test}}$ and Binary${}_{\mathrm{test}}$ is reported in Table 2 (the detailed results of AUC and PRC for each MHC-II molecule were provided in Supplementary Table S4), where the AUCs of the first three compared methods were obtained from their corresponding publications, and that of NetMHCIIpan-4.0_BA was computed from its web server. Here, for the IC50${}_{\mathrm{test}}$ test set, the **Average** indicates the average result of 21 MHC-II molecules each containing more than 20 peptides and at least three binders out of all 47 molecules, and the **All** indicates the result of the whole IC50${}_{\mathrm{test}}$. For the Binary${}_{\mathrm{test}}$ test set, the **Average** indicates the average result of all 10 MHC-II molecules, and the **All** indicates the result of the whole IC50${}_{\mathrm{test}}$. As from Table 2, compared to NetMHCIIpan-3.2, NetMHCIIpan-4.0_BA, PUFFIN, and DeepMHCII, RPEMHC yielded the best prediction results in terms of AUC of Average and All on both independent test sets. Specifically, as compared to NetMHCIIpan-3.2, PUFFIN, and NetMHCIIpan-4.0_BA, RPEMHC performed better on 15, 15, and 14 out of 21 MHC-II molecules on IC50${}_{\mathrm{test}}$, respectively, and on 9, 10, and 9 out of 10 MHC-II molecules on Binary${}_{\mathrm{test}}$, respectively. Additionally, RPEMHC achieved comparable performance against DeepMHCII on both test sets, i.e. better on 11 out of 21 MHC-II molecules and 5 out of 10 MHC-II molecules, respectively. Overall, these results demonstrated the generalization capability of RPEMHC better than those of other state-of-the-art baseline methods on different independent data.

**Table 2.** Comparison of the performance of RPEMHC with NetMHCIIpan-3.2, PUFFIN, DeepMHCII, and NetMHCIIpan-4.0_BA on IC50${}_{\mathrm{test}}$ and Binary${}_{\mathrm{test}}$.^a^

Method	IC50${}_{\mathrm{test}}$		Binary${}_{\mathrm{test}}$	
	AUC		AUC	
	Average	All	Average	All
NetMHCIIpan-3.2	0.733(±0.175)	0.678	0.719(±0.111)	0.719
PUFFIN	0.741(±0.169)	0.688	0.700(±0.105)	0.692
DeepMHCII	0.749(±0.180)	0.693	0.770(±0.103)	0.775
NetMHCIIpan-4.0_BA	0.738(±0.171)	0.681	0.740(±0.082)	0.735
RPEMHC	**0.759(** ± **0.179)**	**0.707**	**0.785(** ± **0.083)**	**0.789**

a The best results are indicated in bold. For metrics in the Average setting, the value in parentheses indicates the standard deviation of all MHC-II molecules.

#### 3.1.4 Independent tests of RPEMHC on T-cell epitope benchmark

Recognizing T-cell epitopes is a difficult task due to the complexity of potentially binding peptides diversity. However, as peptide–MHC binding is a prerequisite for T-cell immunogenicity, many studies have shown that there is a strong correlation between peptide–MHC binding strength and peptide immunogenicity Mustafa and Shaban (2006). Consequently, it is desirable to leverage these peptide–MHC binding affinity prediction methods to recognize T-cell epitopes that match given MHC-II molecules. Given this, we evaluated the capability of RPEMHC on recognizing T-cell epitopes, and compared the prediction performance of RPEMHC to those of NetMHCIIpan3.2, DeepMHCII, and NetMHCIIpan-4.0_BA.

To make a fair comparison with the baseline methods, we followed the annotation method for binders and non-binders in NetMHCIIpan-3.2 and NetMHCIIpan-4.0; specifically, for each pair of MHC-II molecule and epitope, we annotated the epitope as positive and the remaining peptides among all overlapping peptides with the same length as the epitope in the source protein sequence as negatives.

**Figure 5.**
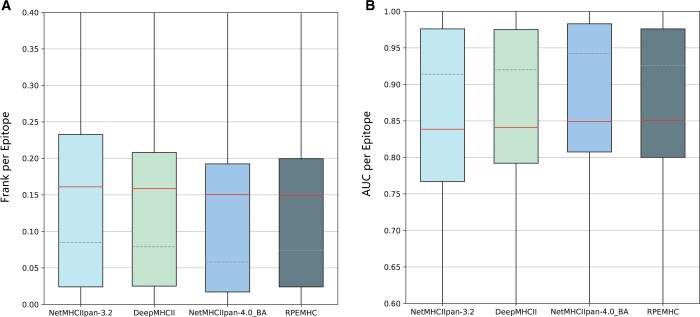
Performance comparison between RPEMHC and NetMHCIIpan-3.2, DeepMHCII, NetMHCIIpan-4.0_BA on T-cell epitope benchmark. (A) The average Frank performance per MHC molecule for NetMHCIIpan-3.2, DeepMHCII, NetMHCIIpan-4.0_BA, and RPEMHC. (B) The average AUC performance per MHC molecule for NetMHCIIpan-3.2, DeepMHCII, NetMHCIIpan-4.0_BA, and RPEMHC.

The performance comparison of RPEMHC with NetMHCIIpan-3.2, DeepMHCII, and NetMHCIIpan4.0_BA on T-cell epitope benchmark in terms of the Frank value and AUC is shown in Fig. 5 (the detailed results of the Frank value and AUC for individual epitopes are provided in Supplementary Table S5), where the results of NetMHCIIpan-3.2 and NetMHCIIpan-4.0_BA were obtained from their web servers, and those of DeepMHCII were computed from the source code implementation under the given parameters. The Frank value refers to the percentage of false-positive predictions within a given epitope source protein, that is, the percentage of peptides with prediction scores higher than that of the positive epitope. An Frank value of 0 corresponds to a perfect prediction namely the positive epitope has the highest predicted binding affinity among all peptides within the source protein, and a value of 0.5 corresponds to the case where an equal number of peptides has a higher and lower prediction value compared with the positive peptide.

As from Fig. 5a, the Frank value of RPEMHC was lower than those of NetMHCIIpan-3.2 and DeepMHCII (paired-samples t-test, *P-*value = $9.355\times 10^{-9}$ and $1.782\times 10^{-6}$, respectively). Moreover, RPEMHC had an average Frank value of 0.149 lower than NetMHCIIpan-3.2 (0.161) and DeepMHCII (0.159), which indicated that the positive peptide was found among the top 15% of the peptides from the source protein if sorted on their predicted peptide binding affinity. Figure 5b also demonstrated an improvement in the AUC performance of RPEMHC against NetMHCIIpan-3.2 and DeepMHCII (paired-samples t-test, *P-*value = $9.566\times 10^{-9}$ and $1.824\times 10^{-6}$, respectively). Additionally, the Frank value of RPEMHC was slightly higher than that of NetMHCIIpan-4.0_BA (paired-samples t-test, *P-*value: $3.361\times 10^{-1}$), but its average Frank value of 0.149 was lower than NetMHCIIpan-4.0_BA (0.150). We speculated that the slight superiority in prediction performance of NetMHCIIpan-4.0_BA could be attributed to the inclusion of EL (Eluted Ligand) data in the training data, which incorporated information not only related to the peptide–MHC binding event but also information about prior steps in the biological antigen presentation pathway processes. Actually, previous studies have also indicated that the integration of BA and EL data for training methods can improve the performance for T-cell epitope prediction compared to methods trained on BA (Binding Affinity) data only Barra *et al.* (2018). With the exception of NetMHCIIpan-4.0_BA, the aforementioned results on three independent tests demonstrated a superior performance of RPEMHC over all other baseline methods trained on BA data only.

#### 3.1.5 The prediction of binding cores

The peptide binding core interacts with the MHC-II binding groove and usually consists of nine amino acids, which primarily determines the MHC-II–peptide binding affinity. Here, we evaluated the performance of RPEMHC on the binding core prediction over the BC2015 dataset, in which the detailed prediction results are shown in Supplementary Table S6. Specifically, for an MHC-II peptide complex, we used a sliding window of size 9 to slide over the peptide sequence to find the amino acid sequence with the highest predicted binding affinity, which is considered to be the binding core of the complex. In comparison with 45 and 47correct predictions out of the 51 peptide binding cores in NetMHCIIpan-3.2 and DeepMHCII, respectively, our method RPEMHC only predicted 31 correct peptide binding cores. However, it can be found from Supplementary Table S6 that among the 20 wrong predictions of the binding cores, most of the cases only appeared 1 or 2 amino acid error offsets.

**Figure 6.**
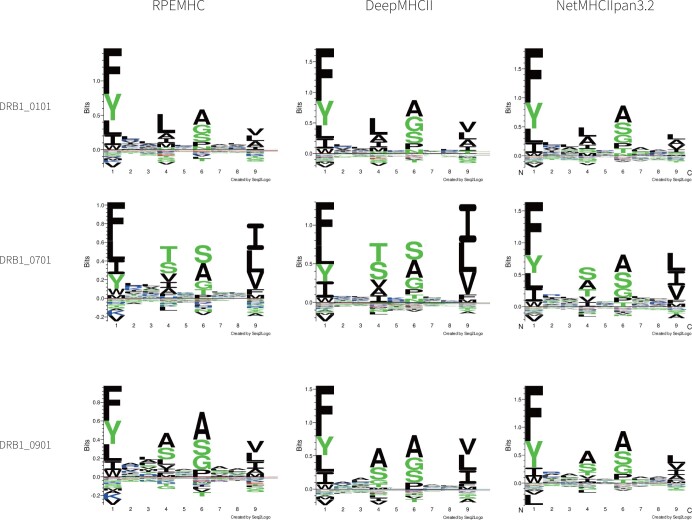
Sequence logos generated by RPEMHC, DeepMHCII, and NetMHCIIpan-3.2. Each sequence logo consists of first to ninth positions in the x-axis, where at each position, the total height of letters (i.e. amino acids) represents the relative information content (also importance) of the corresponding position in the motif, and the height of each letter represents the frequency of the corresponding amino acid in the position.

To further validate the ability of the peptide binding core prediction of RPEMHC, we visualized the binding motifs as sequence logos, which were constructed from the predicted binding cores of the top 1% strongest predicted binders using 100,000 random 15-mer peptides from SwissProt and were visualized using Seq2logo with default settings (Thomsen and Nielsen 2012). We compared the sequence logos generated by RPEMHC, NetMHCIIpan-3.2, and DeepMHCII on eleven MHC-II molecules, each of which contained more than 4000 peptides. For analysis, we show the sequence logos of three molecules DRB1_0101, DRB1_0701, and DRB1_0901 molecules in Fig. 6, and that of all eleven molecules were provided in Supplementary Fig. S1.

From Fig. 6 and Supplementary Fig. S1, it can be observed that the positions 1, 4, 6, and 9 in each sequence logo show more importance. Indeed, these four positions were widely observed as four primary anchors and were most important for MHC-II–peptide binding (Rammensee *et al.* 1999), so it was in accordance with the discovery of primary binding anchors revealed by RPEMHC. Moreover, some differences in the anchoring positions can be observed. For example, for DRB1_0101, the preferred amino acids at position 1 by NetMHCIIpan-3.2, DeepMHCII, and RPEMHC were [LAVISM], [VLAIT], and [VLAI], respectively. According to the MHC binding motif database SYFPEITHI (Rammensee *et al.* 1999), position 1 showed preference for the amino acids [LAIVNFY], so there was no noise in the results of RPEMHC, as compared with the other two methods. For DRB1_0901, SYFPEITHI statistically showed that position 1 showed preference for the amino acids [WYFL], and position 4 showed preference for the amino acids [AVS]. Among the three methods, only the sequence logo generated by RPEMHC contained all the preferred amino acids in SYFPEITHI, while that generated by NetMHCIIpan-3.2 did not contain the amino acid L at position 1, and that generated by DeepMHCII did not contain the amino acid V at position 4. For DRB1_0701, SYFPEITHI showed that position 4 showed preference for the amino acid H, and among the three methods, only the sequence logo generated by RPEMHC contained the amino acid H at position 4. Overall, the four primary anchors can be observed in the sequence logos generated by RPEMHC, DeepMHCII, and NetMHCIIpan3.2, but upon closer inspection, compared to the latter two methods, RPEMHC showed some improvement in the amino acid preferences at some anchoring positions.

### 3.2 Evaluation of the model architecture of RPEMHC generalized to MHC-I–peptide binding

#### 3.2.1 Comparison of RPEMHC with existing methods under five-fold cross-validation on MHC-I_2020_ and MHC-II_2020_

In order to verify the effectiveness of our model architecture, we assessed the prediction capability of RPEMHC on both datasets MHC-I_2020_ and MHC-II_2020_, and compared its prediction performance to those of PUFFIN (Zeng and Gifford 2019) and MHCAttnNet (Venkatesh *et al.* 2020), which were applied to predict MHC–peptide binding for MHC-I and MHC-II molecules. To make a fair comparison, we followed the two baseline methods to perform five-fold cross-validation on these two datasets.

The performance comparison of RPEMHC with PUFFIN, MHCAttnNet on MHC-I_2020_ and MHC-II_2020_ under five-fold cross-validation is shown in Table 3 (the detailed results of five metrics for each MHC-I and MHC-II molecules are provided in Supplementary Tables S7 and S8, respectively), where the results of PUFFIN and MHCAttnNet were obtained from their corresponding publications. Here, the **All** indicates all metrics computed from the whole MHC-I_2020_ and MHC-II_2020_. As seen, RPEMHC outperformed PUFFIN and MHCAttnNet in terms of all metrics except PPV on the prediction of MHC-I and MHC-II–peptide binding. For example, on MHC-I_2020_, RPEMHC achieved improvement in terms of AUC and PCC over PUFFIN by 9.5% and 26.4%, over MHCAttnNet by 2.5% and 4.7%, respectively, and on MHC-II_2020_, over PUFFIN by 3.8% and 5.4%, over MHCAttnNet by 4.9% and 9.2%, respectively. Although our method did not achieve the best on the PPV metric, it is close to that of the best method. Furthermore, we analyzed the performance of RPRMHC on each MHC-I and MHC-II molecule from MHC-I_2020_ and MHC-II_2020_, respectively, as provided in Supplementary Figs. S2 and S3. These results demonstrated that RPEMHC achieved better prediction performance than other baseline methods on the simultaneous prediction of MHC-I and MHC-II–peptide binding.

**Table 3.** Comparison of the performance of RPEMHC with PUFFIN, MHCAttnNet on MHC-I_2020_ and MHC-II_2020_ under five-fold cross-validation.^a^

Method	MHC-I_2020_ (All)			MHC-II_2020_ (All)		
	PUFFIN	MHCAttnNet	RPEMHC	PUFFIN	MHCAttnNet	RPEMHC
AUC	0.819	0.889	**0.914**	0.766	0.755	**0.804**
PCC	0.540	0.757	**0.804**	0.552	0.513	**0.605**
PPV	0.967	**0.968**	0.966	0.864	**0.877**	0.872
Sensitivity	0.721	0.930	**0.940**	0.685	0.730	**0.745**
F1-score	0.826	0.942	**0.953**	0.765	0.768	**0.803**

a The best results are indicated in bold.

#### 3.2.2 Comparison of RPEMHC with existing method under five-fold cross-validation on MHC-I_2015_

In order to further validate the effectiveness of our method RPEMHC on the prediction of MHC-I–peptide binding, we compared the performance of RPEMHC to that of NetMHCpan-3.0 (Nielsen and Andreatta 2016) on the MHC-I_2015_ dataset under five-fold cross-validation. Therefore, NetMHCpan-3.0 was specifically designed for MHC-I–peptide binding.

The average performance comparison between RPEMHC and NetMHCpan-3.0 for different peptide lengths under five-fold cross-validation on MHC-I_2015_ is shown in Table 3 (the detailed results of AUC and PCC for each MHC-I molecule are provided in Supplementary Table S9), where the results of NetMHCpan-3.0 were obtained from its corresponding publication. The **Average** indicates the average result of all MHC-I molecules that contain peptides with the same length. From Table 4, RPEMHC outperformed NetMHCpan-3.0 on peptides of length 9, 10, and 11 (paired-samples t-test, 9-mer: *P-*value = $1.835\times 10^{-3}$ (AUC) and $1.515\times 10^{-4}$ (PCC), 10-mer: *P-*value = $2.716\times 10^{-2}$ (AUC) and $2.294\times 10^{-4}$ (PCC), 11-mer: *P-*value = $4.652\times 10^{-2}$ (AUC) and $4.922\times 10^{-3}$ (PCC)). While for peptides of length 8 and peptides of length 12 or longer, our method RPEMHC performed slightly worse than NetMHCpan-3.0. As a consequence, the above results indicated that our method RPEMHC can be generalized well on the prediction of MHC-I–peptide binding.

**Table 4.** Comparison of the performance of RPEMHC with NetMHCpan-3.0 on peptides of different lengths from MHC-I_2015_ under five-fold cross-validation.^a^

Method	NetMHCpan-3.0 (Average)		RPEMHC (Average)	
	AUC	PCC	AUC	PCC
8-mer	**0.896(** ± **0.072)**	**0.727(** ± **0.117)**	0.892(±0.082)	0.722(±0.127)
9-mer	0.896(±0.086)	0.726(±0.185)	**0.903(** ± **0.080)**	**0.744(** ± **0.165)**
10-mer	0.892(±0.052)	0.761(±0.090)	**0.896(** ± **0.060)**	**0.775(** ± **0.093)**
11-mer	0.885(±0.095)	0.712(±0.128)	**0.897(** ± **0.072)**	**0.731(** ± **0.129)**
≥12-mer	**0.832(** ± **0.130)**	**0.643(** ± **0.189)**	0.822(±0.122)	0.594(±0.245)

a The best results are indicated in bold. For metrics in the Average, the value in parentheses is the standard deviation of all metrics on each MHC molecule.

#### 3.2.3 Independent tests of RPEMHC on CD4 epitope benchmark and CD8 epitope benchmark

We conducted independent validations on two additional sets of T-cell epitope data, i.e. CD4 epitope benchmark and CD8 epitope benchmark, to further evaluate the prediction capability of our method RPEMHC. We also compared PREMHC against NetMHCpan-4.1_BA for class I epitope prediction on the CD8 epitope benchmark, and against NetMHCIIpan-4.0_BA for class II epitope prediction on the CD4 epitope benchmark. The prediction performance for this task was also evaluated in terms of the Frank and AUC values (Supplementary Tables S10 and S11). Specifically, for the CD8 epitope benchmark, RPEMHC achieved a median Frank value of 0.0077, and NetMHCpan-4.1_BA achieved a median Frank value of 0.0067. For the CD4 epitope benchmark, RPEMHC achieved a median Frank value of 0.0494, and NetMHCIIpan-4.0_BA achieved a median Frank value of 0.0434. Consequently, for both class I and II epitope benchmark, RPEMHC was shown to share comparable prediction performance against NetMHCpan-4.1_BA and NetMHCIIpan-4.0_BA. As discussed in Section 3.1.4, a slight drop in the epitope prediction performance of RPEMHC could also be attributed to the inclusion of only BA data in the training data compared to NetMHCpan-4.1 and NetMHCIIpan-4.0 trained including EL data.

### 3.3 Analysis for RPEMHC

#### 3.3.1 Ensemble models

**Figure 7.**
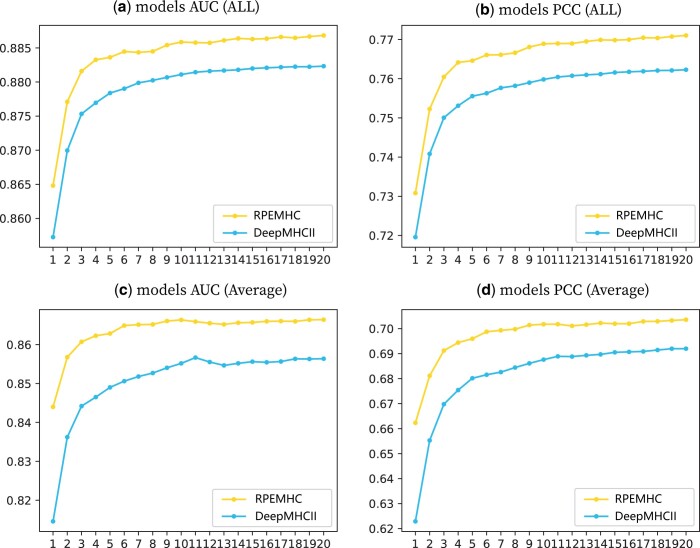
Performance comparison between PREMHC and DeepMHCII with the increase of the number of trained models in the ensemble model measured in terms of AUC and PCC of the whole dataset (a, b), average AUC and PCC (c, d) under five-fold cross-validation on IEDB2016.

We trained RPEMHC independently for twenty times with different random parameters and took the mean value of the twenty experiments as the final prediction value, in order to reduce the randomness of single experiment. The performance comparison between PREMHC and DeepMHCII in terms of AUC and PCC with the increase of the number of trained models in the ensemble model under five-fold cross-validation on the IEDB2016 dataset is shown in Fig. 7, where the results of DeepMHCII were computed from its source code. It can be observed that as the number of trained models in the ensemble model increases, RPEMHC consistently keeps superior to DeepMHCII, and the performance of RPEMHC increased and then remained stable, which demonstrated the role of ensemble models in improving the prediction performance.

#### 3.3.2 Ablation experiments

**Figure 8.**
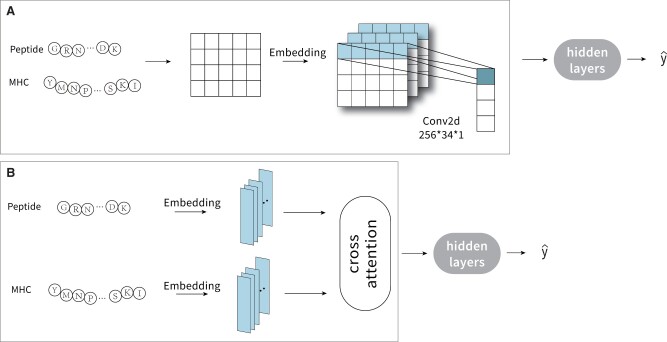
Illustration of the RPE encoding method in the input processing module of RPEMHC (A), and the cross-attention module instead of the input processing module in RPEMHC (B).

In our method RPEMHC, we encoded a pair of an MHC molecule and a peptide into a residue–residue pair map in the input processing module, so as to take advantage of the interaction information between them. Previous work has frequently used the cross-attention mechanism to extract the interaction information between two separate inputs (Vaswani *et al.* 2017, Jin *et al.* 2023). In order to validate the effectiveness of the encoding method of RPE, we replaced the input processing module with the cross-attention module (termed RPEMHC-CA), as shown in Fig. 8. The performance comparison between PREMHC and RPEMHC-CA under five-fold cross-validation on IEDB2016 is shown in Table 5. Apparently, RPEMHC achieved better prediction performance than RPEMHC-CA, which indicated that the RPE encoding method can be more effective in extracting interaction information between MHC molecules and peptides than the cross-attention module.

**Table 5.** Performance comparison between RPEMHC and RPEMHC-CA under five-fold cross-validation on IEDB2016.^a^

Method	Average		All	
	AUC	PCC	AUC	PCC
RPEMHC	**0.866(** ± **0.057)**	**0.704(** ± **0.118)**	**0.887**	**0.771**
RPEMHC-CA	0.843(±0.086)	0.675(±0.141)	0.878	0.757

a The best results are indicated in bold. For metrics in the Average, the value in parentheses is the standard deviation of all metrics on each MHC molecule.

## 4 Conclusion

In this work, we developed a new deep learning method based on residue–residue pair encoding, termed RPEMHC, for the pan-specific prediction of MHC–peptide binding affinity. In particular, RPEMHC encodes a pair of an MHC molecule and a peptide as a residue–residue interaction matrix, in order to capture the critical interaction information between MHC molecules and peptides. Experimental results on a variety of benchmark datasets demonstrated the superior prediction capability of RPEMHC over other state-of-the-art baseline methods on MHC–peptide binding prediction for both MHC-I and MHC-II molecules. In conclusion, these results indicated that RPEMHC is an effective tool for MHC–peptide interaction prediction and may contribute to facilitating the vaccine development.

Some further work that might further improve our method is summarized as follows. First, as previously mentioned in Section 3.2.3, the integration of BA and EL data for training prediction methods can improve their prediction performance, so it is well worthwhile to leverage this integrated datasets to train our method RPEMHC. Second, the large size of the residue–residue pair vocabulary may lead to difficulty for RPEMHC to learn information effectively, and the pairwise residue interaction encoding may lead to misintroduction of interactions that do not exist in some structures. Therefore, screening interactions in structures and exploring more effective ways to encode interactions are deserved to investigate in our future work.

## Supplementary Material

btad785_Supplementary_Data

## Data Availability

No new data were generated or analysed in support of this research.
