# Supplementary material for: RPEMHC: improved prediction of MHC–peptide binding affinity by a deep learning approach based on residue–residue pair encoding
Source: Bioinformatics. 2024 Jan 4;40(1):btad785. doi: 10.1093/bioinformatics/btad785 (PMC10796178; doi:10.1093/bioinformatics/btad785)
Supplement: btad785_Supplementary_Data [file btad785_supplementary_data.zip › RPEMHC_Supplemental Information.docx]

# Supplemental Information

**Text S1: The definition of the five evaluation metrics used in this work.**

**AUC (Area Under the ROC Curve)** measures the ability of a model to distinguish between positive and negative classes by plotting the true positive rate (TPR) against the false positive rate (FPR) at different classification thresholds. The AUC score ranges from 0 to 1, where 1 represents a perfect classifier and 0.5 represents a random guess. A model with an AUC score closer to 1 is considered to have better performance in separating positive and negative instances. It is defined as:

$$AUC = \int_{0}^{1} TPR({FPR}^{-1}(t))dt$$

where TPR is the true positive rate and FPR is the false positive rate, and *FPR^-1^* is the inverse of the cumulative distribution function of the negative class at threshold t.

**PRC (Area Under the PR Curve)** measures the trade-off between the true positive rate and false positive rate for a predictive model by plotting the Precision against the Recall at different classification thresholds. The PRC score ranges from 0 to 1, the higher the PRC score, the better a classifier performs for the given task, where 1 represents a perfect classifier and 0.5 represents a random guess.

**PCC (Pearson correlation coefficient)** is a measure of the linear correlation between two variables, typically used to evaluate the performance of regression models or to assess the relationship between two sets of data. The PCC ranges from -1 to 1, where -1 indicates a perfectly negative linear correlation, 0 indicates no correlation, and 1 indicates a perfectly positive linear correlation. It is defined as:

$$PCC = \frac{\sum_{i-1}^{n} \left( x_{i}-\bar{x} \right)\left( y_{i}-\bar{y} \right)}{\sqrt{\sum_{i-1}^{n} \left( x_{i}-\bar{x} \right)^{2}}\sqrt{\sum_{i-1}^{n} \left( y_{i}-\bar{y} \right)^{2}}}$$

Where x and y are variables, $\bar{x}\mathrm{and}\bar{y}$ are the mean values of x and y, respectively.

**PPV (Positive Predictive Value)** measures the proportion of true positive predictions among all positive predictions made by a model. It is defined as:

$$PPV = {TP}/\left( TP+FP \right)$$

where TP is the number of true positives and FP is the number of false positives. PPV is a useful metric in cases where the cost of a false positive prediction is high. A high PPV indicates that the model is making few false positive predictions, and therefore is highly specific.

**Sensitivity** also known as True Positive Rate (TPR), is a measure of the proportion of actual positive cases that are correctly identified as positive by a model or test. It is calculated as the ratio of true positives to the sum of true positives and false negatives. It is defined as:

$$Sensitivity(TPR) = {TP}/\left( TP+FN \right)$$

where TP represents true positives and FN represents false negatives. Sensitivity is often used in medical diagnosis to measure the ability of a diagnostic test to correctly identify patients with a specific condition.

**F1-score** is a commonly used metric for evaluating the performance of a binary classification model. It is the harmonic mean of precision and recall, and provides a balance between the two. It is defined as:

*F1-score =* $\frac{2\times\left( precision \times recall \right)}{\left( precision + recall \right)}$

**Figure S1** Sequence logos of eleven MHC-II molecules generated by NetMHCIIpan3.2, DeepMHCII (reproduced), and RPEMHC.


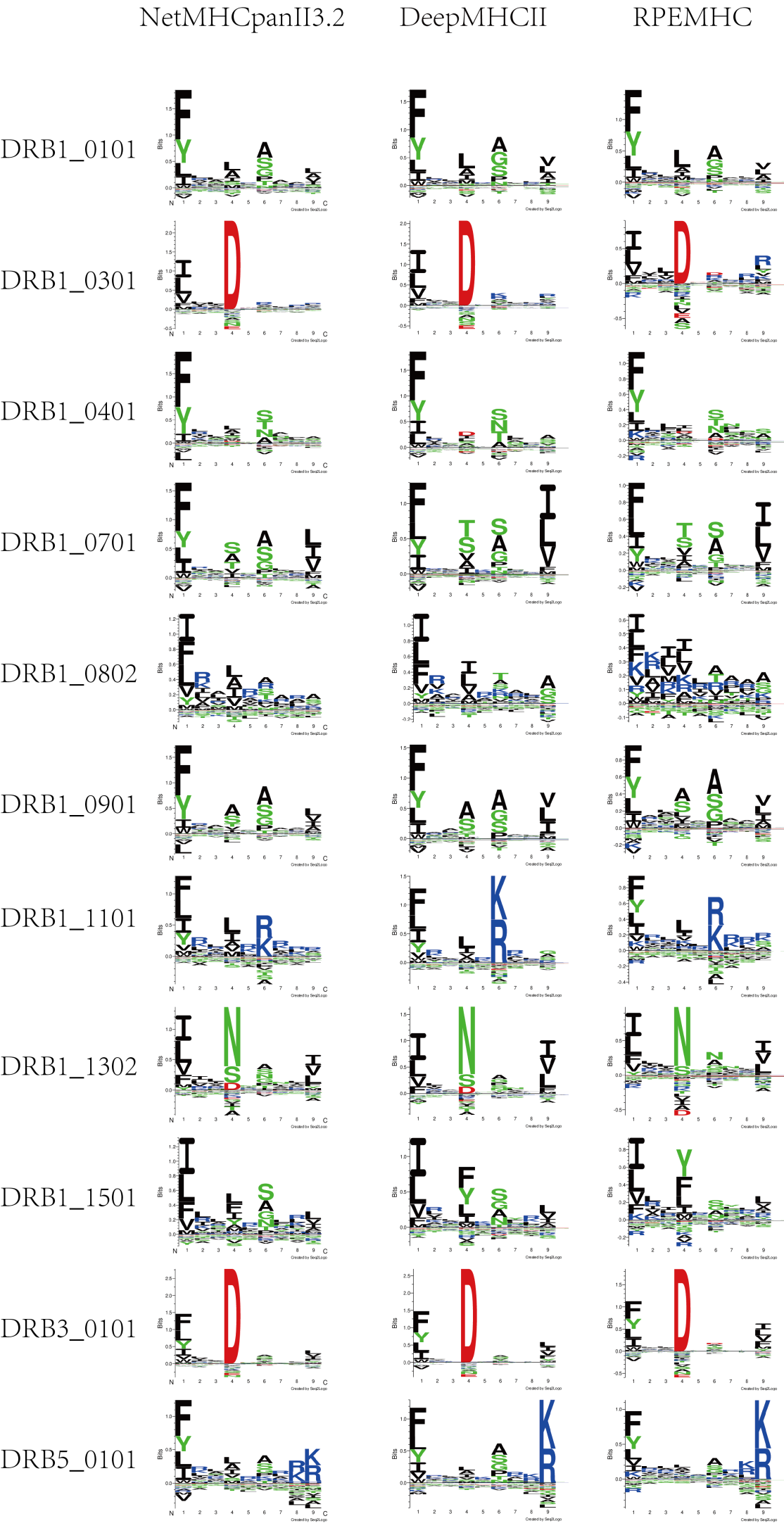


**Figure S2:** Heatmap of AUC, PCC, PVV, Sentivity, and F1-score for 81 MHC-I molecules from MHC-I$_{2020}$. Each column corresponds to an MHC-I molecule.

**
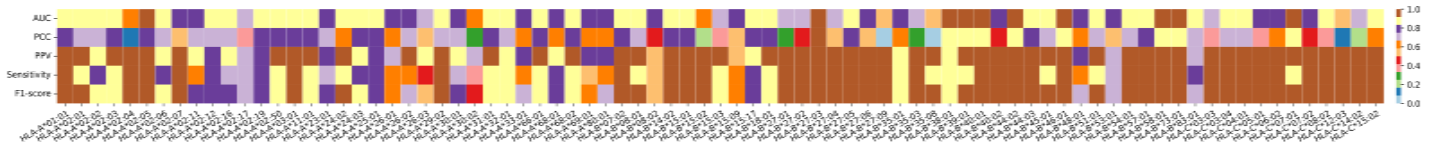
**

**
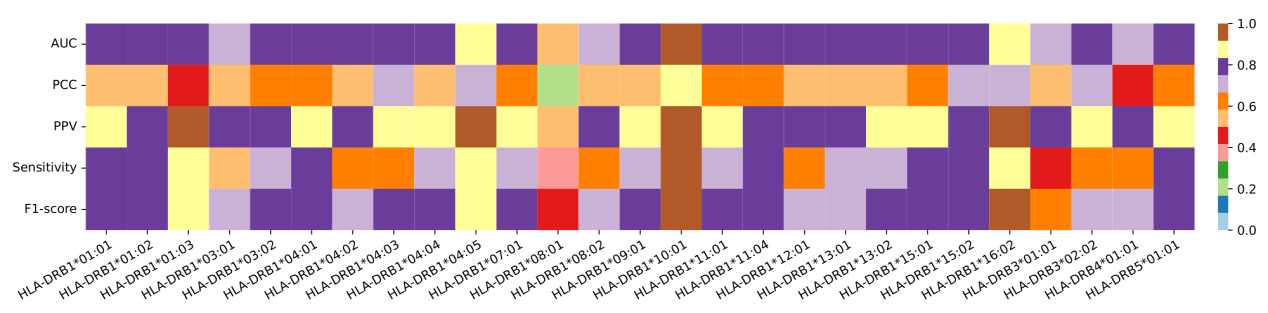
Figure S3:** Heatmap of AUC, PCC, PVV, Sentivity, and F1-score for 27 MHC-II molecules from MHC-II$_{2020}$. Each column corresponds to an MHC-II molecule.
